# Phytoplankton Communities’ Seasonal Fluctuation in Two Neighboring Tropical High-Mountain Lakes

**DOI:** 10.3390/plants13213021

**Published:** 2024-10-29

**Authors:** Rocío Fernández, Javier Alcocer, Luis A. Oseguera, Catriona A. Zuñiga-Ramos, Gloria Vilaclara

**Affiliations:** Grupo de Investigación en Limnología Tropical, FES Iztacala, Universidad Nacional Autónoma de México, Av. de los Barrios 1, Los Reyes Iztacala, Tlalnepantla 54090, Estado de México, Mexico; biol.fernandez@gmail.com (R.F.); loseguera@unam.mx (L.A.O.); catriona19843110@gmail.com (C.A.Z.-R.); vilaclara.gloria@iztacala.unam.mx (G.V.)

**Keywords:** phytoplankton, taxonomic richness, abundance, biomass, alpine lakes, El Sol and La Luna, Nevado de Toluca, Mexico

## Abstract

High-mountain lakes (HMLs) are remote, extreme, and sensitive ecosystems recognized as sentinels of global change. Lakes El Sol and La Luna are very close to each other inside the crater of the Nevado de Toluca volcano, but they differ morphometrically and limnologically. This study aimed to identify the seasonal fluctuation of the phytoplankton communities of these two tropical HMLs. El Sol phytoplankton comprised 50 taxa (chlorophytes, diatoms, charophytes) and La Luna 28 taxa (diatoms, euglenoids). The abundance of phytoplankton in El Sol was three times higher than in La Luna, and the biomass in El Sol was five times higher than in La Luna. Tropical seasonality was reflected differently in each lake. In El Sol, the highest phytoplankton abundance occurred in the rainy season, while the highest biomass was recorded in the dry/warm season. Conversely, in La Luna, abundance and biomass were more prominent in the dry/cold season. The study found that no meteorological or limnological factors could explain the seasonal dynamics of the taxonomic richness, abundance, or biomass of the phytoplankton communities in both lakes. The differences between the lakes are likely due to the more extreme conditions of La Luna, such as lower pH, ultra-oligotrophy, and increased exposure to ultraviolet radiation (UVR). Additionally, the introduction of rainbow trout into El Sol in the 1950s may have also contributed to the differences.

## 1. Introduction

Phytoplankton is the most important primary producer of aquatic ecosystems and plays a primary role in energy flow and carbon cycling [1]. A great deal of research has been carried out to identify the factors influencing their distribution and diversity. Phytoplankton communities respond and adapt to recurring dynamics in aquatic ecosystems [2,3]. Their composition and abundance changes are related to variations in local environmental factors due to seasonality, climate variability, anthropic impacts, and even oceanic–atmospheric phenomena that control global climate [4,5,6].

In an annual cycle, phytoplankton can be controlled by light and nutrient availability, temperature, salinity, and grazing by zooplankton [4]. Adapting phytoplankton to fluctuating environmental factors is crucial for their development or permanence in lakes [3]. Tropical lakes are subject to higher solar radiation intensity and experience lower temperature variation than temperate lakes [7]. Temporal changes in phytoplankton are closely associated with the thermal regime and seasonality [8], which in tropical lakes would be influenced by a notable contrast between rainy and dry seasons [2].

High-altitude areas host numerous lakes, 20% located above 1000 m asl [9]. The phytoplanktonic communities of high-mountain lakes (HML) are adapted to extreme conditions, generally subject to low temperatures, intense solar and ultraviolet (UVR) radiation, limited nutrients, and often acidic pH. UVR is one of the main factors that structure diversity in high-mountain lakes [10,11]. The latter is reflected in the intense pigmentation of zooplankton [12,13], as well as in the phytoplankton community mostly dominated by flagellate organisms [14,15,16] or formation of deep chlorophyll maxima located at depths where phytoplanktonic organisms avoid UVR [9,17].

HMLs are among the most comparable ecosystems in the world [18]. Nonetheless, most information comes from temperate HMLs and only a few from tropical areas [19]. A few recent studies on tropical HMLs have been carried out in the tropical Andean lakes, mainly in Ecuador and Colombia. Recent information on tropical HMLs shows high biodiversity, including phytoplankton, and a multifactorial explanation of the biological dynamics (e.g., [20,21,22,23]). Other papers deal with the adverse effects of anthropogenic activities and climate change on tropical HMLs (e.g., [24,25]).

**Figure 1:**
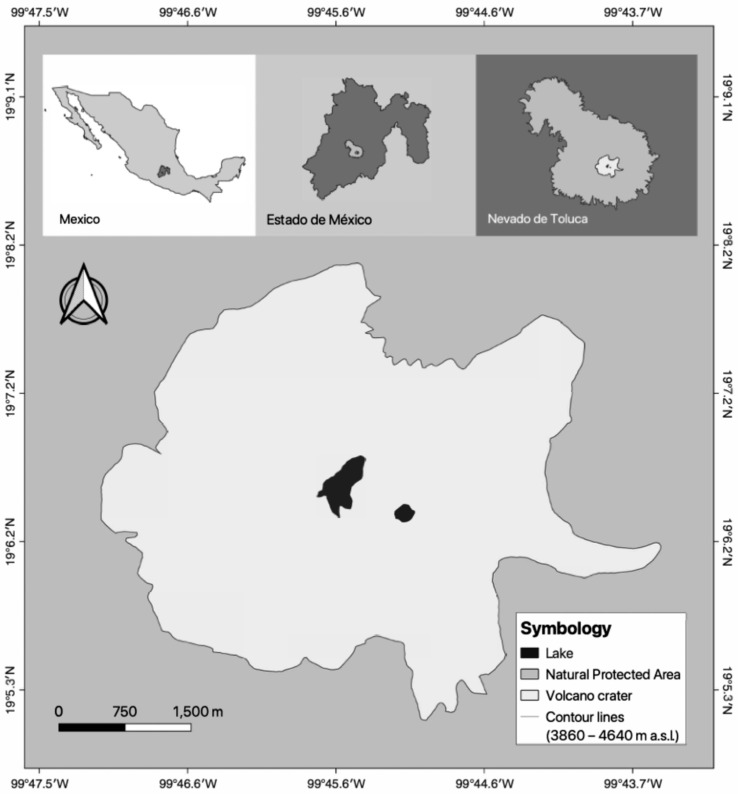
Location of Lakes El Sol and La Luna, inside the crater of the Nevado de Toluca volcano, Estado de México.

The latter leaves a knowledge gap regarding the general limnology and phytoplankton in HMLs in tropical North America. Mexico has just two HMLs, the only HMLs in the expanse between Panama and the northern region of Mexico, at the edge of the tropical region. Lakes El Sol (El Sol from here on) and La Luna (La Luna from here on) are inside the crater of the Nevado de Toluca volcano, Estado de México (19°06′ N, 99°45′ W) at an altitude of 4207 m asl (Figure 1).

El Sol and La Luna are about 600 m apart; they have the same origin, climate, and geology. Despite their proximity and similar environmental context, it has been found [26,27,28] that their physical, chemical, and biological (e.g., macroinvertebrates and zooplankton) characteristics differ. Dimas-Flores et al. [28] explained that the differences in zooplankton between El Sol and La Luna must be related, at least in part, to the introduction of the rainbow trout (*Oncorhynchus mykiss*) in the 1950s.

The present study aims to find whether the effect of tropical seasonality on the dynamics of the phytoplankton communities of these two nearby HMLs can be identified. Tropical seasonality is defined by rain (with a rainy season around summer and dry season around winter), differently from temperate seasonality of higher latitudes, which is determined by temperature and associated with varying rainfall regimes, i.e., a dry summer and wet autumn, winter, and spring. Moreover, Lewis [29] has mentioned that tropical lakes receive higher and less variable solar irradiance than temperate lakes, which differ in the minimum annual irradiance, which is higher in the tropics; the higher solar irradiance largely influences (increases) lake primary productivity and metabolism.

The objectives were to (a) record the seasonal dynamics of the phytoplankton community’s composition, abundance, and biomass in both lakes, (b) identify the principal differences between the phytoplankton communities of both lakes, (c) measure the seasonal dynamics of the main environmental (meteorological and limnological) characteristics of both lakes, and (d) detect the critical environmental variables associated with seasonality of the phytoplankton communities.

## 2. Materials and Methods

### 2.1. Study Area

The Nevado de Toluca is a stratovolcano with a maximum height of 4680 m asl. This andesitic–dacitic volcanic complex is now in a quiescent state; its last eruption occurred about 3300 years BP [30].

The region’s climate is cold, with daily temperature fluctuations of 10 to 20 °C, average maximum temperatures of 20.9 ± 1.4 °C, and an average minimum temperature of 4 ± 1 °C. Annual precipitation is 1277 mm, concentrated from June to September, and the annual evaporation is 971 mm (Station SMN-15062 of the National Meteorological Service). The vegetation inside the crater is sparse and consists of mosses, lichens, and alpine grasses [31].

El Sol has a catchment area of 2.17 km^2^, a surface of 237,321 m^2^, a length of 795 m and a width of 482 m, a perimeter of 2363 m, a maximum depth of 12 m, and an average depth of 6 m. Its thermal regime is discontinuous warm polymictic. It is an oligotrophic lake, with a Secchi disk transparency of 5.4 ± 1.0 m, an average water column temperature of 8.5 ± 1.9 °C, a pH of 7.9 ± 0.2, and an electrical conductivity of 63 ± 20 µS cm^−1^ [27,28,32].

La Luna has a catchment area of 1.1 km^2^, a surface of 30,500 m^2^, a length of 227 m and a width of 209 m, a perimeter of 675 m, a maximum depth of 10 m, and an average depth of 5 m. Its thermal regime is continuously warm polymictic. It is an ultra-oligotrophic lake with a Secchi disk transparency of 9.1 ± 1.8, an average water column temperature of 8.3 ± 2.1 °C, a pH of 4.8 ± 0.1, and an electrical conductivity of 12 ± 3 µS cm^−1^ [27,28,32].

### 2.2. Field Sampling

Meteorological data (daily maximum and minimum temperature, and precipitation) were obtained from the automatic weather station (EMA) at Nevado de Toluca (SMN-CONAGUA, 2023) for the study year. Lake sampling was conducted monthly in one station at each lake, at the central and deepest part, from February 2022 to January 2023. The maximum depth (Z_MAX_) was recorded *in situ*. Water temperature (T), dissolved oxygen concentration (DO), pH, electrical conductivity (K_25_), oxidation-reduction potential (ORP), turbidity (Tur), and chlorophyll-a concentration (Chl-a) were measured *in situ* using a calibrated Hydrolab model DS5 multiparametric probe (vertical resolution 1 m). The number of vertical readings changed according to the maximum depth of each lake at the sampling date. In El Sol, sampling points varied between 10 and 13, and in La Luna, between 8 and 9.

For phytoplankton samples, water was collected with a Niskin bottle. Both lakes are polymictic, so samples were collected at only two depths: 1 m below the surface and 1 m above the bottom. The samples were poured into 600 mL bottles and fixed with Lugol’s acetic iodine solution up to a 1% concentration. Additionally, a vertical drag was carried out along the water column to record those taxa that are not abundant. The drag was concentrated through a 20 µm mesh opening and further fixed with 1% Lugol’s acetic iodine solution up to a 1% concentration.

### 2.3. Laboratory Analysis

The phytoplankton was quantified using the Utermöhl method by sedimenting 50 mL of sample in a sedimentation chamber, with 48 h of settling time [33,34]. Only organisms with cellular content were counted, considering the number of optical fields, until 400 cells of the most abundant species were reached, providing a confidence interval of ± 10% of the mean [34]. The magnification to perform the quantification was selected according to the sample requirements (e.g., higher magnifications for smaller organisms). The number of cells per milliliter was obtained following APHA et al. [35].

The phytoplankton biomass (approximated from cell biovolumes) was obtained by choosing the closest geometric form for each taxon, according to Sun and Liu [36]. The average dimensions needed to calculate the volume of each geometric form were obtained by measuring 20 individuals of each species.

### 2.4. Statistical Analysis

The environmental variables were transformed using the Z-score methodology for statistical analysis, while the logarithm of n + 1 was applied for abundance and biomass. This transformation allowed the data to be normally distributed. One-way analysis of variance (ANOVA) was performed to assess whether there were differences in environmental variables and abundance/biomass of phytoplankton between different climatic seasons. In addition, a Student t-test was performed for independent samples to determine whether there were significant differences between the lakes. When performing a detrended correspondence analysis (DCA) to establish the length of the gradient, a value of 1.254 was obtained, leading to a redundancy analysis (RDA). An RDA was made for each lake, analyzing the environmental variables and the abundance/biomass of phytoplankton species to identify which variable significantly influences the temporal variation of phytoplankton. The statistical significance of RDA was determined by a Monte Carlo permutation test (999), and variable variance inflation factors (VIFs) were calculated to identify possible linear dependencies. A Pearson correlation was used to verify the relationship of environmental variables with biological ones. The statistical analyses were run using RStudio with the Vegan package. The Shannon–Wiener and Pielou evenness indexes were calculated with PAST4 [37].

## 3. Results

### 3.1. Meteorological Variables

**Figure 2:**
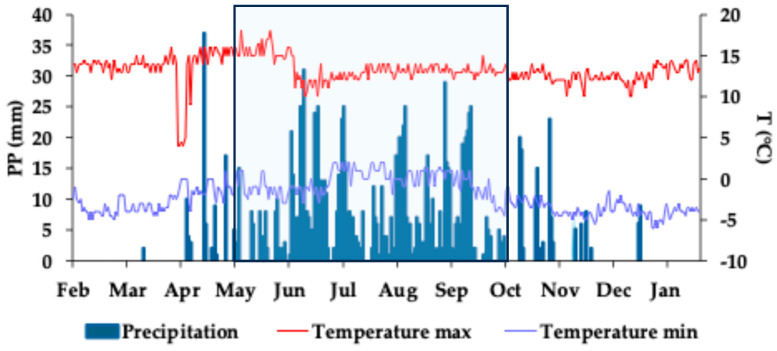
Daily precipitation values and maximum and minimum air temperature in the Nevado de Toluca volcano (EMA weather station Nevado de Toluca: 19.11667 N, −99.76666667 W, 4082 m a.s.l.). The light blue square with a black frame identifies the rainy season.

Precipitation and maximum and minimum daily air temperatures during the study are displayed in Figure 2. According to these variables, three seasons were distinguished: the dry/warm season ranging from February to April and characterized by maximum temperatures of 13.6 ± 2.5 °C, scarce rainfall (0.9 ± 4.3 mm/day), and a daily temperature oscillation of 16.4 ± 3.1 °C; the rainy season from May to September corresponding to the maximum rainy period (6.6 ± 7.7 mm/day) and maximum (13.6 ± 1.5 °C) and minimum daily temperatures (−0.3 ± 1.4 °C) with slight oscillation between them (13.8 ± 2.2 °C); and the dry/cold season from October to January characterized by low temperatures (Tmax 12.6 ±1 °C and Tmin −3 ± 1 °C), rainfall shortages (1.3 ± 4.0 mm/day), and a recorded daily temperature oscillation of 16.4 ± 1.4 °C. Cloudiness and late snow explain the low air temperature for a few days in April, a situation triggered by the effect of a temporary cold front. Nonetheless, this brief period of colder air temperature did not mirror the lakes’ temperature. The increasing water temperature trend in the lakes continued.

Contrasting with the ample daily fluctuations in the air temperature, the lakes’ surface-bottom water temperature differences were reduced, averaging 0.72 ± 0.68 °C in El Sol and 0.28 ± 0.26 °C in La Luna [32]. From this section onwards, the remaining variables are described seasonally (i.e., dry/warm, rainy, dry/cold).

### 3.2. Limnological Variables

Table 1 and Table 2 provide the limnological variables of El Sol and La Luna, respectively. The two lakes are significantly similar (*p* < 0.05) in T, DO, Tur, and ORP and were significantly different (*p* > 0.05) in Z_MAX_, pH, K_25_, and Chl-a. Z_MAX_, pH, K_25_, and Chl-a were higher in El Sol than in La Luna.

Z_MAX_ varied by up to 2.5 m in El Sol and 1.6 m in La Luna. pH in El Sol was higher than in La Luna. In El Sol and La Luna, the pH of the dry/cold season significantly differed (*p* < 0.05) from that of the dry/warm and rainy seasons.

The K_25_ values in El Sol were significantly lower (*p* < 0.05) in the dry/cold season than in the dry/warm and rainy seasons. La Luna also recorded significant seasonal differences (*p* < 0.05), with higher K_25_ values during the rainy season and lower in the dry/warm and dry/cold seasons (Table 1 and Table 2).

In El Sol, Chl-a likewise had significant differences (*p* < 0.05) between the different seasons; the rainy season had higher values than the dry/cold and dry/warm seasons. In La Luna, the highest concentration was recorded in the rainy season, being significantly different (*p* < 0.05) from those of the dry/warm and dry/cold seasons (Table 1 and Table 2).

The lakes displayed different limnological seasonality despite being in the same volcanic basement and exposed to the same climatic characteristics. In El Sol, the dry/warm season presented the highest values of Z_MAX_, K_25_, pH, and ORP and the minimum values of T, Tur, and Chl-a. The rainy season exhibited the highest values of T and Chl-a and the lowest of Z_MAX_ and DO. Finally, in the dry/cold season, the highest values of DO and Tur and the lowest of K_25_, pH, and ORP were measured. In La Luna, the dry/warm season was characterized by the highest values of ORP and the lowest of Z_MAX_, T, K_25_, and Chl-a. The rainy season presented the highest values of T, K_25_, and pH, and the lowest of DO. Finally, in the dry/cold season, the highest values of Z_MAX_, DO, and Chl-a were measured, while the lowest values of pH and ORP were recorded. Turbidity was always minimal, with light reaching the lakes’ bottom.

Even though the limnological variables showed statistically significant differences as described earlier, the differences in the intervals of these variables between the lakes or the seasons are minimal. Therefore, they can be considered to have no or minimal ecological implications. However, it is essential to note that some variables (e.g., pH), especially in La Luna, reach borderline levels for many organisms. In other words, both lakes are considered extreme ecosystems, with La Luna being more extreme than El Sol.

### 3.3. Phytoplankton Composition and Taxonomic Richness

The total phytoplankton taxonomic richness (S) of El Sol and La Luna comprised 63 taxa (Table 3), with 50 taxa in El Sol and 28 taxa in La Luna. Of the 63 taxa, 15 (~24%) were found in both lakes, 33 (~52%) only in El Sol, and 13 (~21%) only in La Luna.

S recorded in El Sol comprised 50 taxa; the most diverse groups of algae were chlorophytes (twelve taxa, 24%), diatoms (seven taxa, 14%), and charophytes (five taxa, 10%). S in La Luna was 28 taxa; the most diverse algal groups were diatoms (six taxa, 21%) and euglenoids (six taxa, 21%) (Table 4).

Only 15 taxa were shared between both lakes: *Aulacoseira* cf. *alpigena*, *Fragilaria crotonensis*, *Pinnularia* cf. *viridis*, *Chromulina* sp., *Golenkinia radiata*, *Oocystis lacustris*, *Cryptomonas* sp., *Limnococcus limneticus*, *Euglena* cf. *variabilis*, *Trachelomonas* cf. *oblonga*, *T*. cf. *planctonica*, *T*. *volvocina*, *Trachelomonas* sp., *Gymnodinium* cf. *lacustre*, and *Parvodinium umbonatum*.

### 3.4. Phytoplankton Abundance and Biomass

**Figure 3:**
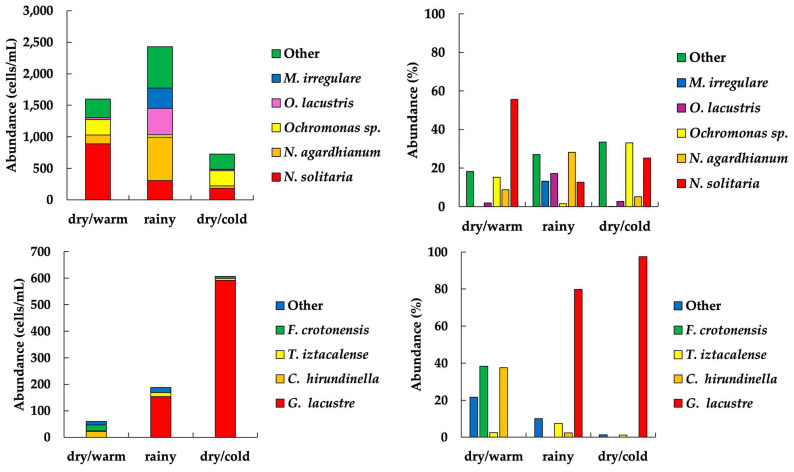
Seasonal variation of the phytoplankton dominant taxa abundance in El Sol (**top**) and La Luna (**bottom**). (Other: comprises the rest of the taxa).

Regarding their contribution to the total abundance, the most important phytoplankton taxa in El Sol were *Oocystis solitaria* (29%), *Nephrocitium agardhianum* (18%), *Ochromonas* sp. 1 (11%), *Oocystis lacustris* (10%), and *Monoraphidium irregulare* (7%) (Figure 3). *Neglectella solitaria* was the most abundant species (138 ± 206 cells/mL), with the highest values during the dry/warm season (299 ± 364 cells/mL). Two to four taxa represented more than 70% of the abundance per season (Table 4, Appendix A). The phytoplankton of La Luna was scarcer than in El Sol. Concerning their contribution to the total abundance, the most important phytoplankton taxa in La Luna were *Gymnodinium* cf. *lacustre* (87%), *Ceratium* cf. *hirundinella* (3%), *Temnogametum iztacalense* (3%), and *Fragilaria crotonensis* (3%) (Figure 3). *Gymnodium* cf. *lacustre* was the most abundant species (124 ± 146 cells/mL), with the highest values during the dry/cold season (197 ± 196 cells/mL). One to two taxa represent more than 75% of the abundance per season (Table 4, Appendix B).

**Figure 4:**
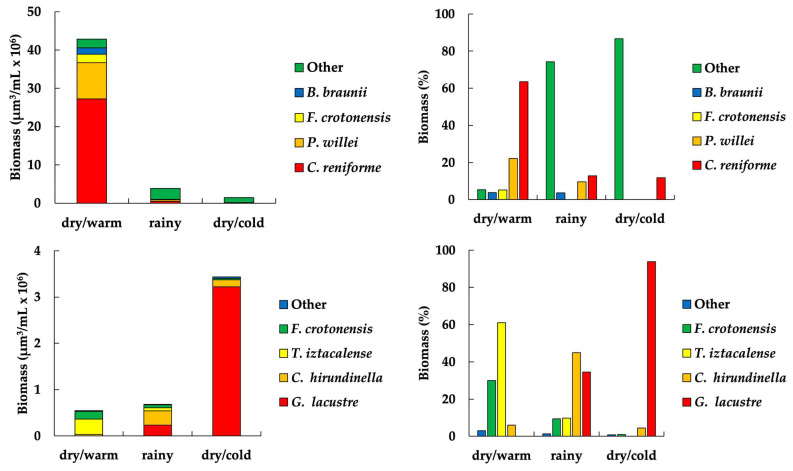
Seasonal variation of the phytoplankton dominant taxa biomass in El Sol (**top**) and La Luna (**bottom**). (Other: comprises the rest of the taxa).

Considering their contribution to biomass, the most important phytoplankton taxa in El Sol were *Cosmarium* cf. *reniforme* (64%), *Peridinium willei* (20%), *Fragilaria crotonensis* (5%), and *Botryococcus braunii* (4%) (Figure 4). *Cosmarium* cf. *reniforme* was the taxon with the highest biomass contribution (2.7 ± 8.6 × 10^6^ µm^3^/L), with its maximum values during the dry/warm season (9.0 ± 15.6 × 10^6^ µm^3^/L). The phytoplankton biomass in El Sol during the dry/warm season ranged from 0.3 to 42.0 × 10^6^ µm^3^/L, corresponding to 84.8% of the total biomass throughout the year; two taxa made up 86% (Figure 4). During the rainy season, biomass represented 11.6% (from 0.5 to 1.5 × 10^6^ µm^3^/L), and 77% comprised six taxa. Finally, during the dry/cold season, the biomass ranged from 0.1 to 0.7 × 10^6^ µm^3^/L, corresponding to 3.5% of the total; two taxa comprised 80% of the biomass (Table 4, Appendix C).

The total biomass of La Luna represents 48% of the total phytoplankton biomass of El Sol. Regarding their contribution to biomass, the most important phytoplankton taxa in La Luna were *Gymnodinium* cf. *lacustre* (74%), *Temnogametum iztacalense* (11%), *Ceratium* cf. *hirundinella* (9%), and *Fragilaria crotonensis* (6%) (Figure 4). The phytoplankton biomass during the dry/warm seasons ranged between 0.09 to 0.3 × 10^6^ µm^3^/mL, corresponding to 11.8% of the total biomass recorded throughout the year; two taxa comprised 86%. During the rainy season, biomass varied from 0.07 to 0.4 × 10^6^ µm^3^/mL (14.7%), where six taxa made up 77% (Figure 4). Finally, in the dry/cold season, the highest biomass values ranged from 0.4 to 2.4 × 10^6^ µm^3^/mL (73.5%); four taxa made up 80% (Table 4, Appendix D).

The higher phytoplankton diversity (H′ = 1.99 ± 0.35) in El Sol compared to La Luna (H′ = 0.86 ± 0.74) suggests a more diverse and balanced ecosystem in El Sol with numerous species (Table 4). However, the moderate H′ and low J′ (0.27 ± 0.05) in El Sol indicate the dominance of a few species. In contrast, the low phytoplankton diversity and J′ (0.33 ± 0.12) in La Luna suggest the dominance of a few species or potential ecological stress (e.g., acidic pH).

### 3.5. Redundancy Analysis and Pearson Correlation

A *p* > 0.05 was observed (Monte Carlo permutation test) in the RDA of both lakes, indicating no correlation between the abundance/biomass of phytoplankton species and the meteorological or limnological variables. The above revealed that any of the variables herein measured would contribute to explaining the phytoplankton dynamics in these HMLs. The Pearson correlations analysis of El Sol revealed water temperature as the most critical variable. The water temperature had a positive correlation with *Monoraphidium irregulare* (*p* = 0.009), *Oocystis lacustris* (*p* = 0.02), and *Ochromonas* sp. 1 (*p* = 0.004). In the analysis of La Luna, no variable had correlations with the abundance or biomass of the phytoplankton community.

## 4. Discussion

The taxonomic richness, S, of El Sol and La Luna is similar to that of other tropical HMLs (see Table 5). The phytoplankton composition of El Sol and La Luna is also comparable to that of other HMLs. In Mexico, diatoms, chlorophytes, and cyanobacteria are the most diverse phytoplankton groups [39,40,41]. Catalan and colleagues [42] noted that mixotrophic organisms (like flagellates from diverse taxonomical groups, *Gymnodinium*, *Chromulina*, *Ochromonas*, *Dinobryon*, and *Cryptomonas*) constitute a significant portion of the phytoplankton in oligotrophic and ultra-oligotrophic lakes. Tolloti and colleagues [15] found that flagellates (chrysophytes, cryptophytes, and dinoflagellates) dominate phytoplankton communities. Flagellated organisms can move deeper into the water column to protect themselves from the high intensity of UV radiation [43,44].

As mentioned at the beginning, HMLs are extreme ecosystems. The lower pH, conductivity, and nutrient concentration in La Luna influence its phytoplankton community’s low values of H′ and J′. El Sol also has low pH, conductivity, and nutrient concentration but is less extreme than La Luna. Thus, its phytoplankton community’s H′ and J′ values are not as low as in La Luna.

Our research underscores the pivotal role of seasonality in shaping the dynamics of phytoplankton communities. For instance, we observed a higher taxonomic richness in the rainy season in both lakes (thirty-one taxa in El Sol and eight taxa in La Luna), with eleven taxa exclusively present during this season. The dominance of certain phytoplankton groups also shifts with the seasons. In both lakes, the dinoflagellates were absent in the rainy season, and the diatoms and cyanobacteria were lacking in the dry/cold period. These findings highlight the intricate and ever-changing interplay of seasonal variations and phytoplankton dynamics, stimulating further exploration.

The abundance and biomass of phytoplankton are associated with dry and rainy periods in tropical inland aquatic ecosystems [45]. The highest abundance and biomass were observed in both lakes and asynchronously in the dry period. The highest peak of abundance and biomass in El Sol was observed in the dry/warm season, while that in La Luna was observed in the dry/cold season. The main species contributing to the abundance are *Neglectella* plus *Oocystis* in El Sol and *Gymnodium* in La Luna; both genera dominate HMLs [46]. Regarding biomass, the prevailing taxa found in El Sol and La Luna (*Cosmarium*, *Fragilaria*, *Peridinium*, *Ceratium*, *Botryococcus*) are frequently reported in HMLs [15,42,45,46,47,48].

The differences in phytoplankton composition, abundance, and biomass in neighboring lakes with similar landscape characteristics are typically linked to geological diversity and morphological features, such as depth, that influence abiotic factors [49,50]. Despite similar climates and geology, El Sol and La Luna differ in shape. El Sol is larger and more profound than La Luna, and the size of its drainage area also differs, with El Sol covering 2.17 km^2^ and La Luna covering 1.1 km^2^. These differences likely account for the varying limnological characteristics (e.g., pH, K_25_, Chl-a) observed in each climatic season (i.e., dry/cold, rainy, dry/warm).

The replacement of phytoplankton taxonomic groups is a well-documented phenomenon, often linked to the dynamics of abiotic and biotic factors such as temperature and pH. For instance, the succession of diatoms is strongly influenced by changes in pH, as noted by Muñoz-López et al. [23]. During our study period, the lowest pH values (El Sol: 7.1 ± 0.1, La Luna: 3.9 ± 0.1) were recorded in the dry/cold season. Ibarra-Morales et al. [51] found that the atmospheric bulk deposition reaching Nevado de Toluca varies between the cold/dry season with SW–NE wind direction and the warm/rainy season with NE–SW wind direction. In the warm/rainy season, pH and K_25_ were lower than in the cold/dry season. The more acidic bulk deposition during the warm/rainy season explains the lower pH values recorded in the lakes during this season. Ibarra-Morales [pers. com., October 2024] found no difference between the bulk deposition in El Sol and La Luna; therefore, the differences in pH and other variables between El Sol and La Luna could be associated, at least partially, with the larger area draining into El Sol.

Environmental stress reduces diversity, allowing only species tolerant of that stress to dominate [4]. An example is *Temnogametum iztacalense* in La Luna, a green alga (Charophyta, Zygnematatophyceae) closely related to *Mougeotia*. This well-known acidophilic species indicates low pH values in the lake (lower than El Sol). This green alga’s intense purple color also reflects a protective mechanism against the high UV radiation that reaches the bottom of this shallow and transparent lake.

On the other hand, the convergence of multiple factors results in stochastic or non-linear responses in the phytoplankton community [46,52]. In this sense, although a more significant variation was observed in the phytoplankton community than in the limnological factors, the meteorological variables’ seasonality seemed to drive the dynamics of limnological factors, both in El Sol (T, pH, K_25_, Chl-a) and La Luna (pH, K_25_, ORP).

The lower abundance and biomass of phytoplankton in Lake La Luna compared to Lake El Sol could be linked to the more extreme conditions in La Luna, such as its ultra-oligotrophy and very low pH and K_25_, as well as its shallowness and high water transparency. These conditions induce high doses of ultraviolet radiation (UVR) to reach the bottom of the lake, providing no depth refuge for plankton protection. The significance of low pH and high UVR incidence were suggested to be the most critical factors in reducing plankton diversity, as indicated by Dimas-Flores et al. [28] for the zooplankton of Lake La Luna.

In the 1950s, the Mexican government introduced rainbow trout (*Oncorhynchus mykiss*) into the lakes, a species that only remains in El Sol. The lack of baseline studies conducted before the fish’s introduction makes it impossible to fully understand the extent of the modification it caused. However, their introduction is a stark reminder of the potentially significant impact of human interventions on natural ecosystems. The differences between the two lakes, with higher pH, nutrient concentration, primary productivity, turbidity, etc., in El Sol, can be partly attributed to this introduction. It is also expected that the introduction of the trout modified the original biological composition and abundance of El Sol by introducing exotic species along with the trout.

The less extreme conditions of El Sol allow for more abundant phytoplankton [the present study] and zooplankton [28] communities than in La Luna; the abundance in La Luna is 1/5th in phytoplankton and 1/100th in zooplankton that of El Sol. Zooplankton predation pressure on phytoplankton must be higher in El Sol than in La Luna. However, this has neither been evaluated nor has its implications on phytoplankton dynamics been considered, indicating an opportunity for future studies.

## 5. Conclusions

El Sol and La Luna were shown to be limnologically different, mainly in pH, K_25_, and Chl-a concentration, with higher values in El Sol. The tropical seasonality is expressed in three climatic seasons (dry/cold, rainy, and dry/warm) and mirrored in the lakes’ seasonally different environmental characteristics (e.g., T, K_25_, pH, Chl-a). Despite finding statistically significant differences in some limnological variables between the two lakes, the ranges of the values for these variables are very similar between both lakes and climate seasons. Therefore, it is likely that these differences are not ecologically relevant. Despite this fact, El Sol and La Luna’s phytoplankton communities differed.

The taxonomic richness of El Sol’s phytoplanktonic community comprised 50 taxa, while La Luna’s included only 28. The most diverse phytoplankton groups were chlorophytes, diatoms, and charophytes in El Sol, and diatoms and euglenoids in La Luna. *Neglectella solitaria* and *Cosmarium* cf. *reniforme* dominated in abundance and biomass, respectively, in El Sol. *Gymnodium* cf. *lacustre* dominated in both abundance and biomass in La Luna. As expected, no statistical correlations were found between the phytoplankton communities and the limnological variables.

The different taxonomic composition and dominance, and the lower values of S, abundance, and biomass in La Luna compared to El Sol, are probably associated with (1) the different morphometry (e.g., depth, length) and the size of its drainage areas (i.e., more extensive in El Sol), (2) the more extreme conditions of La Luna (e.g., lower pH, ultra-oligotrophy, increased exposure to UVR), and (3) the introduction of rainbow trout that undoubtedly altered the original limnological characteristics of El Sol.

Regarding future endeavors, paleolimnological evidence could help us understand the original characteristics of El Sol, its resemblance to La Luna, and the importance of the morphometric differences between both lakes. Furthermore, it could provide insights into the impact of introducing rainbow trout on the limnology of El Sol and the speed at which these changes occurred. This evidence would also contribute to our understanding of the effects of introducing exotic species on pristine HMLs.

## Figures and Tables

**Table 1:** Environmental variables (average ± standard deviation) of Lake El Sol, Nevado de Toluca, measured during February 2022–January 2023. (Z_MAX_ = maximum depth, T = temperature, K_25_ = electrical conductivity, ORP = oxidation-reduction potential, DO = dissolved oxygen, Tur = turbidity, Chl-a = chlorophyll-a concentration).

Variable	Dry/Warm	Rainy	Dry/Cold	Annual
Z_MAX_ (m)	10.8 ± 0.9	9.8 ± 1.0	10.3 ± 0.3	10.3 ± 0.7
T (°C)	7.8 ± 0.2	11.2 ± 0.3	7.9 ± 0.2	9.0 ± 0.2
K_25_ (µS/cm)	45.3 ± 0.2	42.8 ± 0.3	39.1 ± 0.5	42.4 ± 0.3
pH	8.5 ± 0.1	8.3 ± 0.3	7.1 ± 0.1	7.9 ± 0.2
ORP (mV)	384 ± 4	311 ± 16	284 ± 3	326 ± 8
DO (mg/L)	7.9 ± 0.1	6.0 ± 1.2	8.1 ± 0.8	7.3 ± 0.7
Tur (NTU)	0.0 ± 0.0	0.1 ± 0.3	0.2 ± 0.4	0.1 ± 0.2
Chl-a (µg/L)	0.4 ± 0.0	1.3 ± 0.2	1.0 ± 0.0	0.9 ± 0.9

**Table 2:** Environmental variables (average ± standard deviation) of Lake La Luna, Nevado de Toluca, measured during February 2022–January 2023. (Z_MAX_ = maximum depth, T = temperature, K_25_ = electrical conductivity, ORP = oxidation-reduction potential, DO = dissolved oxygen, Tur = turbidity, Chl-a = chlorophyll-a concentration).

Variable	Dry/Warm	Rainy	Dry/Cold	Annual
Z_MAX_ (m)	7.2 ± 0.8	7.3 ± 0.6	7.4 ± 0.4	7.3 ± 0.6
T (°C)	8.3 ± 0.1	11.1 ± 0.2	8.3 ± 0.5	9.2 ± 0.2
K_25_ (µS/cm)	9.6 ± 0.1	10.5 ± 0.1	10.1 ± 0.1	10.0 ± 0.1
pH	5.0 ± 0.1	5.6 ± 0.2	3.9 ± 0.1	4.8 ± 0.1
ORP (mV)	467 ± 11	384 ± 22	381 ± 10	410 ± 14
DO (mg/L)	6.9 ± 0.0	6.3 ± 0.0	7.2 ± 0.0	6.8 ± 0.0
Tur (NTU)	0.0 ± 0.0	0.0 ± 0.0	0.0 ± 0.0	0.0 ± 0.0
Chl-a (µg/L)	0.2 ± 0.0	0.3 ± 0.0	0.5 ± 0.0	0.3 ± 0.0

**Table 3:** Phytoplankton taxonomic list of El Sol and La Luna, Nevado de Toluca, México. Nomenclature follows that of AlgaeBase (H.R. = higher ranks, phylum and class) [38]: H = Heterokontophyta [Hd, diatoms; Hc, chrisophytes], ChZ = Charophyta Zygnematophyceae, Cl = Clorophyta [ClT, Trebouxiophyceae; ClC, Chlorophyceae; ClP, Pyramimonadophyceae], Cr = Cryptista Cryptophyceae, Cy = Cyanobacteria, E = Euglenophyta, D = Dinoflagellata. X indicates the presence of taxa, while - indicates their absence).

N°	H.R.	Taxon	El Sol	La Luna
1	Hd	*Aulacoseira* cf. *alpigena*	X	X
2	Hd	*Aulacoseira nivaloides*	X	-
3	Hd	*Cyclotella* sp.	X	-
4	Hd	*Cymbella* sp.	-	X
5	Hd	*Fragilaria crotonensis*	X	X
6	Hd	*Frustulia rhomboides*	-	X
7	Hd	*Navicula* sp.	X	-
8	Hd	*Pinnularia* cf. *viridis*	X	X
9	Hd	*Pinnularia subcapitata*	-	X
10	Hd	*Synedra* cf. *ulna*	X	-
11	Hc	*Chromulina* sp.	X	X
12	Hc	*Chrysococcus minutus*	X	-
13	Hc	*Dinobryon* cf. *sociale*	X	-
14	Hc	*Mallomonas* cf. *akrokomos*	X	-
15	Hc	*Ochromonas* sp. 1	X	-
16	Hc	*Ochromonas* sp. 2	-	X
17	ChZ	*Closterium setaceum*	X	-
18	ChZ	*Cosmarium* cf. *ocellatum*	X	-
19	ChZ	*Cosmarium* cf. *reniforme*	X	-
20	ChZ	*Staurastrum* cf. *gracile*	X	-
21	ChZ	*Staurastrum* sp.	X	-
22	ChZ	*Temnogametum iztacalense*	-	X
23	ClT	*Botryococcus braunii*	X	-
24	ClT	*Chlorella* sp.	-	X
25	ClT	*Crucigeniella* sp.	X	-
26	ClT	*Neglectella solitaria*	X	-
27	ClT	*Oocystis lacustris*	X	X
28	ClC	*Chlorogonium minimum*	X	-
29	ClC	*Gemellicystis planctonica*	X	-
30	ClC	*Gloeocystis* sp.	X	-
31	ClC	*Golenkinia radiata*	X	X
32	ClC	*Kirchneriella lunaris*	X	-
33	ClC	*Kirchneriella obesa*	X	-
34	ClC	*Microglena monadina*	X	-
35	ClC	*Monoraphidium griffithii*	X	-
36	ClC	*Monoraphidium irregulare*	X	-
37	ClC	*Nephrocitium agardhianum*	X	-
38	ClC	*Palmella* sp.	X	-
39	ClC	*Pediastrum simplex*	-	X
40	ClP	*Pyramimonas* sp.	X	-
41	Cr	*Cryptomonas* sp.	X	X
42	Cy	*Anabaena* sp.	X	-
43	Cy	*Anabaenopsis* sp.	-	X
44	Cy	*Gomphosphaeria* sp.	X	-
45	Cy	*Limnococcus limneticus*	X	X
46	Cy	*Merismopedia elegans*	-	X
47	Cy	*Microcystis* cf. *botrys*	X	-
48	Cy	*Pseudanabaena* sp.	X	-
49	Cy	*Synechococcus* sp.	X	-
50	Cy	*Synechocystis* sp.	-	X
51	E	*Euglena* cf. *variabilis*	X	X
52	E	*Euglena* sp.	-	X
53	E	*Lepocinclis* cf. *ovum*	X	-
54	E	*Trachelomonas* cf. *oblonga*	X	X
55	E	*Trachelomonas* cf. *planctonica*	X	X
56	E	*Trachelomonas* sp.	X	X
57	E	*Trachelomonas volvocina*	X	X
58	D	*Ceratium* cf. *hirundinella*	-	X
59	D	*Gymnodinium* cf. *lacustre*	X	X
60	D	*Gymnodinium lantzschii*	-	X
61	D	*Parvodinium umbonatum*	X	X
62	D	*Peridinium* cf. *volzii*	X	-
63	D	*Peridinium willei*	X	-

**Table 4:** Average taxonomic richness (S), number of exclusive taxa per season, abundance and biomass of phytoplankton, number of dominant taxa per season, and the Shannon–Wiener diversity (H′) and Pielou evenness (J′) indexes in El Sol and La Luna.

**El Sol**	**Dry/Warm**	**Rainy**	**Dry/Cold**	**Annual**
S	12.0 ± 2.6	14.9 ± 3.6	13.3 ± 7.0	13.6 ± 4.3
Exclusive spp.	4	8	7	-
Abundance (cells/mL)	532.5 ± 414.9	607.3 ± 286.0	242.2 ± 12.3	475.3 ± 304.1
Ab. Dominant spp.	2 (71%)	4 (71%)	4 (74%)	5 (75%)
Biomass (µm^3^/mL)	14.18 ± 24.17 × 10^6^	0.95 ± 0.41 × 10^6^	0.49 ± 0.32 × 10^6^	4.81 ± 13.14 × 10^6^
Bio. Dominant spp.	2 (86%)	6 (77%)	4 (80%)	2 (78%)
H′	1.61	2.27	2.10	2.44
J′	0.22	0.30	0.30	0.23
**La Luna**	**Dry/Warm**	**Rainy**	**Dry/Cold**	**Annual**
S	5.3 ± 0.6	2.9 ± 0.3	3.0 ± 1.0	3.7 ± 1.3
Exclusive spp.	5	3	1	-
Abundance (cells/mL)	20.0 ± 6.3	46.9 ± 52.2	202.3 ± 200.8	85.5 ± 128.5
Ab. Dominant spp.	2 (76%)	1 (80%)	1 (98%)	1 (87%)
Biomass (µm^3^/mL)	0.18 ± 0.16 × 10^6^	0.17 ± 0.16 × 10^6^	1.04 ± 0.94 × 10^6^	0.44 ± 0.62 × 10^6^
Bio. Dominant spp.	2 (91%)	2 (79%)	1 (94%)	2 (85%)
H′	1.63	0.81	0.15	0.68
J′	0.46	0.28	0.23	0.12

**Table 5:** Phytoplankton taxonomic richness (S), abundance (Ab), biomass (Bio), and dominant group (Dom) in tropical HMLs. (Ref = reference: 1: [21], 2: [19], 3: [25], 4: [20], 5: Present research).

Site	Lake	S	Ab (Cell/mL)	Bio (µm^3^/mL)	Dom	Ref
Colombian Andes	Sol	20			Charophyta (49 taxa),	1
	Frailejones	48			Heterokontophyta (diatoms (23 taxa),	
	Los Tutos	42			Cyanobacteria (20 taxa),	
	Verde	24			Chlorophyta (19 taxa),	
	Pozo Verde	31			Heterokontophyta (chrysophytes (5 taxa), Dinoflagellata (4 taxa)	
	San Pablo	31	4000		Chlorophyta (16 taxa), Heterokontophyta (diatoms,6taxa)	2
Ecuadorian Andes	Paramo Lakes (11 lakes)	12 to 45		2 to 4000	Chlorophyta (60%), dinoflagellates (18%), Euglenophyta (10%)	3
	Glacial Lakes (5 lakes)	13 to 24		0 to 400	Cryptista (50%), Dinoflagellata (32%)	
	Condorshillu, Tres Lagunas, and Laguna Grande	15 to 43	4066 to 120,914	3.63 to 241.67 × 10^6^	Bacillariophyta (50%), Dinoflagellata (24%)	4
Nevado de Toluca	El Sol	50	475.3 ± 304.1	4.81 ± 13.14 × 10^6^	Chlorophyta (16 taxa, 32%), Heterokontophyta (diatoms, 7 taxa, 14%; chrysophytes, 5 taxa, 10%), Euglenophyta (6 taxa, 12%), Charophyta (5 taxa, 10%), Dinoflagellata (4 taxa, 8%), Cyanobacteria (6 taxa, 12%)	5
	La Luna	28	85.5 ± 128.5	0.44 ± 0.62 × 10^6^	Chlorophyta (4 taxa, 14%), Heterokontophyta (diatoms, 6 taxa, 21%; chrysophytes, 2 taxa, 7%), Euglenophyta (6 taxa, 21%), Charophyta (1 taxa, 4%), Dinoflagellata (4 taxa, 14%), Cyanobacteria (4 taxa, 8%)	

## Data Availability

Data are available from the authors upon reasonable request.

## Appendix A

**Table A1:** Abundance (cells/mL) of the different phytoplankton taxa recorded in El Sol.

Taxon	Dry/Warm			Rainy			Dry/Cold		
*Aulacoseira* cf. *alpigena*	1.5	±	2.2	0.5	±	1.0	0.0	±	0.0
*Aulacoseira nivaloides*	2.5	±	4.3	0.0	±	0.0	0.0	±	0.0
*Cyclotella* sp.	0.0	±	0.0	6.5	±	13.0	0.0	±	0.0
*Fragilaria crotonensis*	12.7	±	15.2	0.0	±	0.0	0.0	±	0.0
*Navicula* sp.	0.5	±	0.9	0.0	±	0.0	0.0	±	0.0
*Pinnularia* cf. *viridis*	0.2	±	0.3	0.0	±	0.0	0.0	±	0.0
*Synedra* cf. *ulna*	0.7	±	1.2	0.9	±	1.8	0.0	±	0.0
*Chromulina* sp.	0.7	±	1.2	0.0	±	0.0	0.0	±	0.0
*Chrysococcus minutus*	0.0	±	0.0	0.0	±	0.0	5.3	±	9.2
*Dinobryon* cf. *sociale*	0.0	±	0.0	0.0	±	0.0	0.2	±	0.3
*Mallomonas* cf. *akrokomos*	0.0	±	0.0	0.0	±	0.0	9.2	±	15.9
*Ochromonas* sp. 1	81.2	±	57.2	9.8	±	12.2	80.3	±	94.4
*Closterium setaceum*	0.5	±	0.9	0.0	±	0.0	22.3	±	27.8
*Cosmarium* cf. *ocellatum*	0.2	±	0.3	0.0	±	0.0	0.5	±	0.5
*Cosmarium* cf. *reniforme*	2.8	±	4.5	1.4	±	0.9	0.7	±	0.8
*Staurastrum* cf. *gracile*	0.0	±	0.0	0.0	±	0.0	0.3	±	0.6
*Staurastrum* sp.	0.0	±	0.0	0.3	±	0.5	0.2	±	0.3
*Botryococcus braunii*	3.3	±	5.8	2.8	±	5.5	0.0	±	0.0
*Crucigeniella* sp.	0.0	±	0.0	26.1	±	32.8	0.0	±	0.0
*Neglectella solitaria*	296.0	±	363.9	76.9	±	52.8	61.2	±	18.4
*Oocystis lacustris*	10.7	±	16.8	104.9	±	99.9	6.5	±	7.7
*Chlorogonium minimum*	2.7	±	2.5	0.0	±	0.0	16.0	±	16.3
*Gemellicystis planctonica*	0.0	±	0.0	4.1	±	8.3	0.0	±	0.0
*Gloeocystis* sp.	0.0	±	0.0	4.1	±	5.9	0.0	±	0.0
*Golenkinia radiata*	0.3	±	0.5	0.0	±	0.0	0.0	±	0.0
*Kirchneriella lunaris*	0.0	±	0.0	0.0	±	0.0	0.2	±	0.3
*Kirchneriella obesa*	0.0	±	0.0	1.0	±	2.0	0.0	±	0.0
*Microglena monadina*	0.0	±	0.0	0.0	±	0.0	2.8	±	4.9
*Monoraphidium griffithii*	19.8	±	17.3	12.9	±	20.0	1.8	±	3.2
*Monoraphidium irregulare*	0.0	±	0.0	80.5	±	95.0	0.3	±	0.6
*Nephrocitium agardhianum*	47.2	±	58.5	171.1	±	213.6	12.7	±	21.9
*Palmella* sp.	0.0	±	0.0	0.4	±	0.8	0.0	±	0.0
*Pyramimonas* sp.	0.0	±	0.0	0.5	±	1.0	0.0	±	0.0
*Cryptomonas* sp.	0.0	±	0.0	8.9	±	16.1	10.8	±	11.2
*Anabaena* sp.	32.8	±	40.1	1.3	±	2.5	0.0	±	0.0
*Gomphosphaeria* sp.	0.0	±	0.0	33.8	±	67.5	0.0	±	0.0
*Limnococcus limneticus*	0.0	±	0.0	0.6	±	0.9	0.2	±	0.3
*Microcystis* cf. *botrys*	0.0	±	0.0	35.3	±	56.2	0.0	±	0.0
*Pseudanabaena* sp.	3.8	±	6.6	0.0	±	0.0	0.7	±	1.2
*Synechococcus* sp.	0.0	±	0.0	0.0	±	0.0	0.8	±	1.4
*Euglena* cf. *variavilis*	0.3	±	0.6	4.6	±	6.0	0.8	±	1.0
*Lepocinclis* cf. *ovum*	0.0	±	0.0	0.5	±	1.0	2.3	±	4.0
*Trachelomonas* cf. *oblonga*	8.7	±	15.0	1.5	±	1.2	0.2	±	0.3
*Trachelomonas* cf. *planctonica*	0.0	±	0.0	0.3	±	0.5	0.0	±	0.0
*Trachelomonas* sp.	0.0	±	0.0	0.0	±	0.0	3.2	±	3.0
*Trachelomonas volvocina*	2.5	±	4.3	2.6	±	1.8	0.0	±	0.0
*Gymnodinium* cf. *lacustre*	0.0	±	0.0	0.6	±	0.8	0.7	±	1.2
*Parvodinium umbonatum*	0.0	±	0.0	9.4	±	18.8	2.0	±	3.5
*Peridinium* cf. *volzii*	0.0	±	0.0	2.3	±	4.5	0.0	±	0.0
*Peridinium willei*	1.3	±	2.3	1.3	±	1.0	0.0	±	0.0

## Appendix B

**Table A2:** Abundance (cells/mL) of the different phytoplankton taxa recorded in La Luna.

Taxon	Dry/Warm			Rainy			Dry/Cold		
*Aulacoseira* cf. *alpigena*	0.7	±	1.2	3.1	±	6.3	0.0	±	0.0
*Cymbella* sp.	0.0	±	0.0	0.03	±	0.05	0.0	±	0.0
*Fragilaria crotonensis*	7.7	±	4.8	0.0	±	0.0	0.0	±	0.0
*Frustulia rhomboides*	0.0	±	0.08	0.0	±	0.0	0.0	±	0.0
*Pinnularia* cf. *viridis*	0.2	±	0.3	0.0	±	0.0	0.0	±	0.0
*Pinnularia subcapitata*	0.0	±	0.0	0.1	±	0.3	0.0	±	0.0
*Chromulina* sp.	0.0	±	0.0	0.2	±	0.3	0.0	±	0.0
*Ochromonas* sp. 2	0.2	±	0.3	0.0	±	0.0	0.0	±	0.0
*Temnogametum iztacalense*	0.6	±	0.4	3.5	±	3.6	2.3	±	2.0
*Chlorella* sp.	0.0	±	0.0	0.1	±	0.2	0.0	±	0.0
*Oocystis lacustris*	0.8	±	0.8	0.0	±	0.0	0.0	±	0.0
*Golenkinia radiata*	0.0	±	0.0	0.2	±	0.3	0.0	±	0.0
*Pediastrum simplex*	0.0	±	0.0	0.03	±	0.1	0.0	±	0.0
*Cryptomonas* sp.	0.3	±	0.6	0.0	±	0.0	0.0	±	0.0
*Anabaenopsis* sp.	1.7	±	2.9	0.0	±	0.0	0.0	±	0.0
*Limnococcus limneticus*	0.0	±	0.0	0.1	±	0.3	0.8	±	1.4
*Merismopedia elegans*	0.3	±	0.6	0.0	±	0.0	0.0	±	0.0
*Synechocystis* sp.	0.0	±	0.0	1.1	±	2.3	0.0	±	0.0
*Euglena* cf. *variabilis*	0.01	±	0.01	0.2	±	0.2	0.0	±	0.0
*Euglena* sp.	0.2	±	0.3	0.0	±	0.0	0.0	±	0.0
*Trachelomonas* cf. *oblonga*	0.3	±	0.6	0.0	±	0.0	0.5	±	0.5
*Trachelomonas* cf. *planctonica*	0.1	±	0.1	0.1	±	0.1	0.0	±	0.0
*Trachelomonas* sp.	0.0	±	0.0	0.0	±	0.0	1.3	±	2.3
*Trachelomonas volvocina*	0.3	±	0.6	0.0	±	0.0	0.0	±	0.0
*Ceratium* cf. *hirundinella*	7.5	±	13.0	1.1	±	2.3	0.0	±	0.0
*Gymnodinium* cf. *lacustre*	0.0	±	0.0	37.5	±	50.6	197.3	±	196.5
*Gymnodinium lantzschii*	0.0	±	0.0	0.3	±	0.5	0.0	±	0.0
*Parvodinium umbonatum*	0.0	±	0.0	0.4	±	0.6	0.0	±	0.0

## Appendix C

**Table A3:** Biomass (µm^3^/mL) of the different phytoplankton taxa recorded in El Sol.

Taxon	Dry/Warm			Rainy			Dry/Cold		
*Aulacoseira* cf. *alpigena*	7067	±	1027	236	±	471	0	±	0
*Aulacoseira nivaloides*	2985	±	5170	0	±	0	0	±	0
*Cyclotella* sp.	0	±	0	18,369	±	36,738	0	±	0
*Fragilaria crotonensis*	751,136	±	1,248,554	0	±	0	0	±	0
*Navicula* sp.	1848	±	3201	0	±	0	0	±	0
*Pinnularia* cf. *viridis*	52	±	91	0	±	0	0	±	0
*Synedra* cf. *ulna*	277,348	±	480,381	4116	±	8232	0	±	0
*Chromulina* sp.	223	±	387	0	±	0	0	±	0
*Chrysococcus minutus*	0	±	0	0	±	0	2392	±	4142
*Dinobryon* cf. *sociale*	0	±	0	0	±	0	92	±	159
*Mallomonas* cf. *akrokomos*	0	±	0	0	±	0	2127	±	3684
*Ochromonas* sp. 1	34,814	±	21,856	1912	±	2397	15,755	±	18,524
*Closterium setaceum*	6581	±	11,399	0	±	0	275,343	±	376,373
*Cosmarium* cf. *ocellatum*	14,818	±	25,666	0	±	0	4903	±	4903
*Cosmarium* cf. *reniforme*	9,069,413	±	15,670,200	122,251	±	75,921	59,273	±	67,906
*Staurastrum* cf. *gracile*	0	±	0	0	±	0	2376	±	4115
*Staurastrum* sp.	0	±	0	2646	±		1764	±	3056
*Botryococcus braunii*	546,954	±	947,351	35,077	±	70,155	0	±	0
*Crucigeniella* sp.	0	±	0	1968	±	2470	0	±	0
*Neglectella solitaria*	267,588	±	215,573	129,100	±	88,687	102,720	±	30,878
*Oocystis lacustris*	34,311	±	58,850	35,071	±	33,476	2176	±	2576
*Chlorogonium minimum*	9256	±	15,798	0	±	0	1440	±	1463
*Gemellicystis planctonica*	0	±	0	138	±	276	0	±	0
*Gloeocystis* sp.	0	±	0	7281	±	10,402	0	±	0
*Golenkinia radiata*	1185	±	2053	0	±	0	0	±	0
*Kirchneriella lunaris*	0	±	0	0	±		49	±	85
*Kirchneriella obesa*	0	±	0	2388	±	4776	0	±	0
*Microglena monadina*	0	±	0	0	±	0	6335	±	10,972
*Monoraphidium griffithii*	9466	±	8240	6145	±	9530	875	±	1516
*Monoraphidium irregulare*	0	±	0	5957	±	7030	25	±	43
*Nephrocitium agardhianum*	47,999	±	7282	73,883	±	92,209	5469	±	9472
*Palmella* sp.	0	±	0	13	±	25	0	±	0
*Pyramimonas* sp.	0	±	0	177	±	353	0	±	0
*Cryptomonas* sp.	0	±	0	68,701	±	124,834	83,860	±	86,315
*Anabaena* sp.	24,598	±	38,568	283	±	565	0	±	0
*Gomphosphaeria* sp.	0	±	0	4793	±	9585.0	0	±	0
*Limnococcus limneticus*	0	±	0	49	±	74	13	±	23
*Microcystis* cf. *botrys*	0	±	0	147	±	235	0	±	0
*Pseudanabaena* sp.	96	±	167	0	±	0	17	±	29
*Synechococcus* sp.	0	±	0	0	±	0	146	±	252
*Euglena* cf. *variabilis*	1401	±	2427	19,442	±	25,068	3503	±	4375
*Lepocinclis* cf. *ovum*	0	±	0	714	±	1429	3334	±	5774
*Trachelomonas* cf. *oblonga*	27,903	±	48,330	4829	±	3943	537	±	929
*Trachelomonas* cf. *planctonica*	0	±	0	602	±	1205	0	±	0
*Trachelomonas* sp.	0	±	0	0	±	0	17,529	±	16,683
*Trachelomonas volvocina*	1740	±	3014	1827	±	1251	0	±	0
*Gymnodinium* cf. *lacustre*	0	±	0	3400	±	4079	3626	±	6281
*Parvodinium umbonatum*	0	±	0	118,692	±	237,384	25,321	±	43,857
*Peridinium* cf. *volzii*	0	±	0	201,648	±	403,296	0	±	0
*Peridinium willei*	3,152,143	±	5,459,671	91,888	±	70,381	0	±	0

## Appendix D

**Table A4:** Biomass (µm^3^/mL) of the different phytoplankton taxa recorded in La Luna.

Taxon	Dry/Warm			Rainy			Dry/Cold		
*Aulacoseira* cf. *alpigena*	471	±	666	222,312	±	444,624	0	±	0
*Cymbella* sp.	0	±	0	298	±	597	0	±	0
*Fragilaria crotonensis*	55,016	±	34,477	16,146	±	32,292	10,764	±	15,640
*Frustulia rhomboides*	0	±	0	4333	±	8666	0	±	0
*Pinnularia* cf. *viridis*	63	±	109	0	±	0	0	±	0
*Pinnularia subcapitata*	0	±	0	544	±	1088	0	±	0
*Chromulina* sp.	0	±	0	54	±	107	0	±	0
*Ochromonas* sp. 2	33	±	57	0	±	0	0	±	0
*Temnogametum iztacalense*	113,188	±	7925	76,930	±	79,756	51,287	±	44,416
*Chlorella* sp.	0	±	0	5	±	10	0	±	0
*Oocystis lacustris*	1400	±	1283	0	±	0	0	±	0
*Golenkinia radiata*	0	±	0	4333	±	8666	0	±	0
*Pediastrum simplex*	0	±	0	191	±	383	0	±	0
*Cryptomonas* sp.	2580	±	4469	0	±	0	0	±	0
*Anabaenopsis* sp.	353	±	612	0	±	0	0	±	0
*Limnococcus limneticus*	0	±	0	29	±	59	131	±	226
*Merismopedia elegans*	1	±	2	0	±	0	0	±	0
*Synechocystis* sp.	0	±	0	127	±	254	0	±	0
*Euglena* cf. *variabilis*	28	±	48	736	±	867	0	±	0
*Euglena* sp.	1127	±	1953	0	±	0	0	±	0
*Trachelomonas* cf. *oblonga*	1073	±	1859	0	±	0	1610	±	1610
*Trachelomonas* cf. *planctonica*	160	±	278	120	±	241	0	±	0
*Trachelomonas* sp.	0	±	0	0	±	0	7381	±	12,784
*Trachelomonas volvocina*	232	±	402	0	±	0	0	±	0
*Ceratium* cf. *hirundinella*	223,650	±	387,373	33,548	±	67,095	0	±	0
*Gymnodinium* cf. *lacustre*	0	±	0	59,003	±	65,036	107,3336	±	1,068,711
*Gymnodinium lantzschii*	0	±	0	339	±	678	0	±	0
*Parvodinium umbonatum*	0	±	0	4115	±	6649	0	±	0
